# Major Systemic Lupus Erythematosus Exacerbation after Severe Clostridium Difficile Infection: A Case Report

**DOI:** 10.31138/mjr.190224.msl

**Published:** 2024-12-31

**Authors:** Styliani Partalidou, Ioanna Katsigianni, Vasiliki Tara, Elpiniki Retzeperi, Anastasios Radounislis, Ioannis Eleftherios Neofytou, Ioannis Valsamidis, Anthimos Pehlivanidis

**Affiliations:** 1 First Department of Internal Medicine;; 2 Rheumatology Department;; 3 Nephrology Department, 424 General Military Hospital of Thessaloniki, Thessaloniki, Greece

**Keywords:** lupus flare, clostridium difficile infection, lupus nephritis, serositis, case report

## Abstract

**Introduction:**

Systemic lupus erythematosus (SLE) is a multisystem autoimmune disease presenting with remission and flares. Relapses may be triggered by various factors, with infections being one of the most common. The following case is the first clostridium difficile infection (CDI)-induced SLE flare that resulted in involvement of organs not previously affected in patient’s history before, such as lupus nephritis.

**Case presentation:**

We present a case of a 77-year-old woman, who experienced a major flare, involving renal impairment, cardiorespiratory deterioration and pleuritis, along with signs of haemolytic anaemia, three weeks after a severe CDI. She received corticosteroids, rituximab (RTX), and cyclophosphamide (CYC), but the outcome was still fatal.

**Conclusion:**

CDI infections are highly increasing in frequency and severity, given the antibiotic tolerance, so clinicians should bear in mind the risk of immune-mediated disorders reactivation.

## INTRODUCTION

Systemic lupus erythematosus (SLE) is a multisystem autoimmune disease, affecting mainly, women of child-bearing age.^1^ Its usual manifestations include low grade fever, arthralgia of small and large joints and hematologic abnormalities, such as lymphopenia, anaemia, and/or thrombocytopenia. Additionally, many patients present with kidney-related symptoms, including proteinuria, haematuria, elevated blood pressure, and renal impairment with elevated creatinine levels.^2^ In fact, many studies suggest that 40% to 70% of SLE patients will develop some degree of kidney involvement during disease course, often within five years of initial presentation.^3^

SLE is a B-cell driven autoimmune disorder, and the plethora of auto-antibodies is the serological hallmark of the disease.^4^ This immunological dysregulation constitutes patients with SLE prone to infections, common or opportunistic. Inversely, infections, may also lead to an SLE flare. In fact, infections are considered to be one of the leading death causes in this population.^5^ As more and more advanced antibiotic regimens are used in real-life clinical practice, along with the exacerbation of hospital-acquired infections by multi-resistant bacteria, clostridium difficile infection (CDI) incidence seems to rise.^6^ Here, we present a case of a major SLE flare in an elderly patient with recent severe CDI.

## CASE PRESENTATION

### First admission

We present a case of a 77-year-old woman who was admitted to our hospital due to fever and multiple episodes of diarrhoea, while she was under post-surgery antibiotic therapy, following a total hip replacement, conducted for advanced osteoarthritis. Despite the rehabilitation period, the patient was capable of self-care. Furthermore, her mental status was intact. The medical history of the patient was notable for SLE, which started forty years ago, managed adequately with low dose of corticosteroids (GCs), namely 2mg methylprednisolone and hydroxychloroquine (HCQ) 200mg twice a day. The rest of her medical history included arterial hypertension and paroxysmal atrial fibrillation. The SLE manifestations she had experienced at disease onset included diffuse arthralgias, arthritis of wrists, leukopenia, and pleuritis. The family history was unremarkable.

At presentation the patient was orientated, febrile at 38 degrees Celsius, her blood pressure was 120/80mmHg and the saturation was 90% while breathing ambient air. The physical examination revealed no abnormal findings. A faecal specimen came back positive for toxin A of Clostridium difficile, a finding compatible with recently used antibiotics. The laboratory findings were notable for high inflammation indexes, mainly leucocytosis, high C-reactive protein (CRP), and procalcitonin, while her renal function was within normal range.

The patient was treated with standard of care, with metronidazole and vancomycin. Due to the severity of infection the GCs were withdrawn in order to prevent immunosuppression, while HCQ was preserved. The patient responded well, both clinically and serologically in about seven days, with remission of diarrhoea and fever. A few days after improvement, she presented mild increase of the body temperature, in a range of 37.1–37.3 degrees of Celsius, despite having negative blood and urine cultures, negative flu and COVID-19 rapid antigen tests and no other findings indicative of infection. At the same time, she began experiencing pain and oedema in the wrists, knees and feet. Laboratory findings revealed mild increase of CRP, high erythrocyte sedimentation rate (ESR) and low complement levels. As these findings were suggestive of possible SLE flare, we initiated 4mg methylprednisolone. The patient, again responded efficiently and her clinical symptoms resolved, leading to her discharge three days later.

### Second admission

Three weeks later, the patient presented again in the Emergency Department complaining about vomiting episodes, one or two per day, as well as anaemia, low calcium and potassium levels detected during regular laboratory check, so the patient was admitted for further investigation. The clinical examination revealed no abnormal clinical findings. An endoscopy of upper gastrointestinal track was performed with unremarkable findings. As the trials with domperidone and ondansetron were ineffective, a magnetic resonance imaging (MRI) was conducted, and central causes of vomit were excluded. At the same time, dexamethasone was initiated, at a dose of 8mg twice a day intravenously.

The patient’s creatinine levels then gradually increased over the course of a week, peaking at 3mg/dl, accompanied by normal to high potassium levels. The patient sustained adequate diuresis (with the occasional use of loop diuretics), did not experience any hypotensive episodes and remained clinically hydrated. Urgent computed tomography (CT) of the abdomen was conducted to rule out occlusive causes, yielding negative results. Additionally, the patient had not been exposed to any apparent nephrotoxic agent, received appropriate maintenance fluids and exhibited elevated systolic blood pressure ranging from 150 to 170mmHg. Despite diligent investigation, no obvious cause for this acute renal injury could be identified. Urine analysis was, also, positive for protein exertion and the 24-hour urine revealed 1gr of proteinuria.

**Figure 1.**
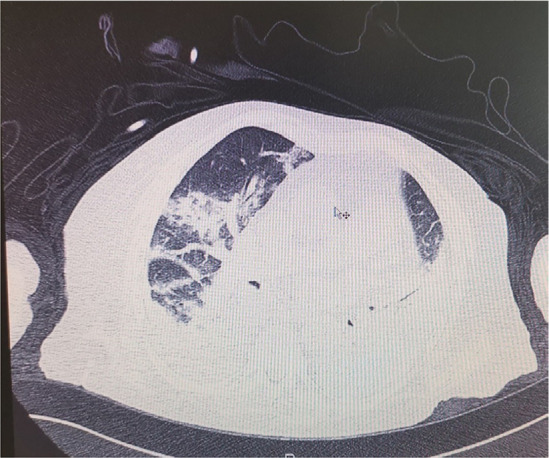
Chest CT scan.

**Figure 2.**
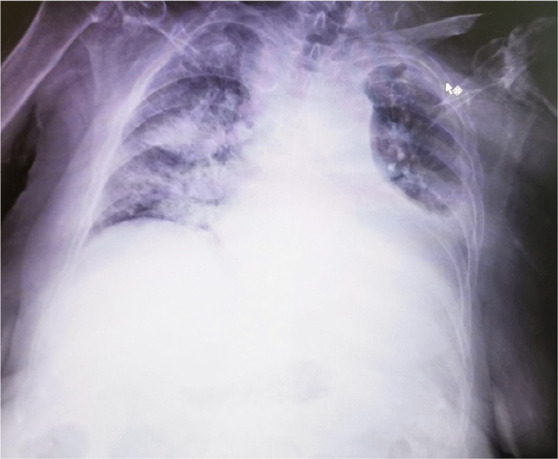
Chest X-ray.

The patient began to be agitated at first, and then disorientated and unable to speak articulately. An emergency head CT did not appeal any acute cerebrovascular event. The dexamethasone dose was reduced, and the patient’s mental status improved. Apart from the above-mentioned signs and symptoms, the patient also had laboratory findings suggestive of haemolytic anaemia (low haemoglobin, positive direct Coombs test, high reticulocyte number and high levels of lactate dehydrogenase), elevated troponin levels with diastolic dysfunction and pulmonary hypertension in the heart ultrasound, as well as rapid progression of pleuritis-more pronounced at the left, and bilateral ground-glass opacities, with exceeded needs of high flow oxygenation. **Figures 1 and 2** present a chest CT scan and X-Ray respectively.

**Figure 3.**
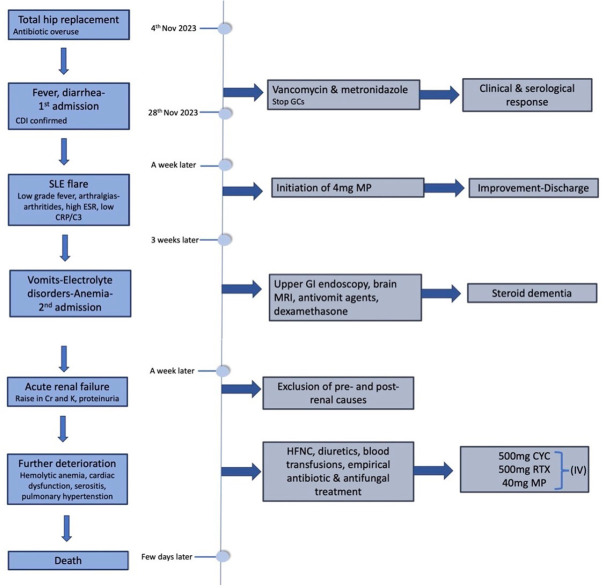
Timeline of patient’s disease course. CDI: clostridium difficile infection; Cr: creatinine; CRP: C-reactive protein; CYC: cyclophosphamide; ESR: erythrocyte sedimentation rate; GCs: glucocorticoids; GI: gastrointestinal; HFNC: high flow nasal canula; K: potassium; MP: methylprednisolone; RTX: rituximab; SLE: systemic lupus erythematosus.

All the above clues indicated that our patient was experiencing a severe flare of SLE, incorporating major systems, with nephritis, haemolytic anaemia, cardiac dysfunction, and serositis. Due to respiratory instability, renal biopsy was not performed, preventing a definitive diagnosis from being reached. It was then decided to initiate immunosuppression therapy, so she received 500mg of cyclophosphamide (CYC), followed by 500mg of rituximab (RTX), along with methylprednisolone of 40mg intravenously. At first, she seemed to respond adequately, exhibiting respiratory improvement (shrinkage of serositis, less oxygen needs), elevation of haemoglobin levels, as well as gradual improvement of creatinine levels. However, soon enough, the patient developed fever spikes, despite empirical antibiotic and antifungal treatment (1gr meropenem three times per day and 100mg fluconazole twice a day intravenously), decreased consciousness and respiratory rebound, and she passed away three days later. Cultures revealed a urinary tract infection caused by Gram negative bacteria, sensitive to the empirical antibiotic treatment she was already receiving. A timeline of patient’s disease course is provided in **Figure 3**.

## DISCUSSION

SLE is a chronic multisystem disorder, presenting with periods of remission alternating with periods of relapses. Flares may be triggered by various reasons, such as infection, ultraviolet radiation, hormonal changes as they happen in pregnancy, and drugs, and they are usually observed within approximately four years after a complete or partial response. 7,8 Also, renal flares are more common in patients who had already experienced lupus nephritis earlier in the disease course.^9^ However, literature review reveals reports of patients with late-onset lupus nephritis, which was also associated -among others- with respiratory involvement.^10,11^ Assuming that our patient’s renal manifestations were SLE-related, given the absence of a better explanation, it is noteworthy that this was her first renal presentation, after a long-lasting, mainly mild, disease course.

According to European Alliance of Associations for Rheumatology (EULAR) guidelines for SLE, we treated the patient with CYC, as suggested for life-threatening organ-involvement. Due to the rapid progression of the disease, along with patient’s comorbidities, an RTX infusion was additionally implemented without delay in the therapeutic plan in order to rapidly regain major organ functionality.^12^

Another clinical challenge in this case was, also, the differential diagnosis of central nervous system (CNS) symptoms, namely agitation, hallucinations, and speech difficulties, as they could be incorporated in the clinical setting of SLE flare. However, the patient had never before CNS manifestations, and, also, this clinical picture appeared few days after the initiation of high doses of dexamethasone. CNS side effects of GCs, particularly high doses, are well described in the literature, and they can include psychosis, confusion, insomnia, depression, memory dysfunction, as well as cognitive deficits, known as «steroid dementia».^13,14^ Eventually, we concluded that this was a steroid-induced situation, as the symptomatology improved soon after GC dose reduction.

Many hypotheses regarding the possible etiopathogenetic role of gut microbiota and various autoimmune diseases, such as SLE have been made.^15,16^ To date, ***Ruminococcus gnavus*** presents the strongest correlation according to a case-control study. SLE patients had the microorganism five times more frequently in their gut microbiota, and its detection also correlated with higher disease activity and the presence of lupus nephritis.^17^ Moreover, in a recent review, Charoensappakit et al. analyse how various gut infections can harm mucosa barrier. This leads to the dispersion of pathogen molecules in the bloodstream, which in turn may cause an SLE exacerbation, as a result of the immunological response to these signals.^18^

One of our approach’s strengths is that it underlines the rising hazard of CDI, not only as a severe infection itself, but also, as a significant trigger for immune-mediated disease reactivation, incorporating manifestations that were never observed before in a particular patient. On the other hand, our clinical strategy did not lack limitations, as well. As highlighted above, we did not conduct a renal biopsy, given the cardiorespiratory instability of the patient, as long as with the rapid deterioration, needing immediate intervention.

## CONCLUSION

To the best of our knowledge, this is the first case reported in the literature presenting CDI as a possible causable factor of a severe SLE relapse, resulting in fatal outcome. The link between gut infections and SLE pathogenesis, as well as trigger for flares needs further elucidation in a clinical setting.
